# Treatment success and mortality among adults with tuberculosis in rural eastern Uganda: a retrospective cohort study

**DOI:** 10.1186/s12889-020-08646-0

**Published:** 2020-04-15

**Authors:** Jonathan Izudi, Imelda K. Tamwesigire, Francis Bajunirwe

**Affiliations:** Department of Community Health, Faculty of Medicine, Mbarara University of Science and Technology, P.O. Box, 1410, Mbarara, Uganda

**Keywords:** HIV, Mortality, Treatment success rate, Tuberculosis, Uganda

## Abstract

**Background:**

Successful treatment of tuberculosis leads to clinical and public health benefits such as reduction in transmission, complications, and mortality among patients. However, data are limited on treatment outcomes and the associated factors among persons with bacteriologically confirmed pulmonary (BC-PTB) in rural areas of high dual tuberculosis and Human Immunodeficiency Virus (HIV) burden countries such as Uganda. We investigated factors associated with successful treatment of tuberculosis and mortality among adult persons with BC-PTB in rural eastern Uganda.

**Methods:**

We constructed a retrospective cohort of persons with BC-PTB from a routine tuberculosis clinic database in eastern Uganda. We performed bivariate and multivariate analysis. Using a 5% level of significance, we ran a modified Poisson regression analysis to determine factors independently associated with treatment success and mortality rates.

**Results:**

We retrieved 1123 records for persons with BC-PTB and the treatment outcomes were distributed as follows: 477(42.5%) cured, 323 (28.0%) treatment completed, 17(1.5%) treatment failed, 81(7.2%) died, 89(7.9%) lost to follow-up, and 136(12.1%) not evaluated. Overall, 800 (81.1%) of the 987 persons with BC-PTB that had treatment outcome, were successfully treated. Successful treatment of tuberculosis was less likely to occur among those with HIV infection (Adjusted risk ratio (aRR), 0.88; 95% Confidence Interval (CI), 0.82–0.95), older than 50 years (aRR, 0.89; 95% CI, 0.81–0.97), or male sex (aRR, 0.92; 95% CI, 0.87–0.98). Mortality was associated with HIV infection (aRR, 4.48; 95% CI, 2.95–6.79), older than 50 years (aRR, 2.93; 95% CI, 1.74–4.92), year of enrollment into treatment after 2015 (aRR, 0.80; 95% CI, 0.66–0.97), and Community-Based Directly Observed Therapy Short Course (aRR, 0.26; 95% CI, 0.13–0.50).

**Conclusions:**

Treatment success rate among adult persons with BC-PTB in rural eastern Uganda is suboptimal and mortality rate is high. HIV infection and older age reduce chances of treatment success, and increase mortality rate. Older and HIV infected persons with BC-PTB will require special consideration to optimize treatment success rate and reduce mortality rate.

## Background

Although tuberculosis is treatable and curable with a standard course of antibiotics, the disease continues to claim millions of lives globally. The 2019 World Health Organization (WHO) Global Tuberculosis report indicates that 10 million people developed tuberculosis disease in 2018 and 1.5 million of them died [1]. The WHO recommends that a good performing tuberculosis program should achieve at least 90% treatment success rate and 85% cure rate [2]. These targets contribute to the effective reduction of tuberculosis transmission at household and community levels, and in reducing tuberculosis related complications and mortality [3]. Nonetheless, tuberculosis control programs all over the world have challenges in meeting the recommended treatment success rate particularly for persons with new bacteriologically confirmed pulmonary tuberculosis (BC-PTB) diagnosis. According to current data, global treatment success rate for persons with new BC-PTB diagnosis improved from 82% in 2016 [4] to 85% in 2017 [1], which is still lower than the desired target of at least 90%.

Sub Saharan Africa has the highest burden of tuberculosis and the slowest decline in the number of tuberculosis incident cases [5], and suboptimal tuberculosis treatment success rate. Recent systematic review and meta-analysis show a treatment success rate of 76.2% among persons with BC-PTB in sub Saharan Africa over the past 10 years [6], far below the global treatment success rate of 85% [1] and the WHO recommended rate of at least 90% [2]. This meta-analysis also shows that sub Saharan Africa may be experiencing a gradual but steady decline in tuberculosis treatment success rate [6]. There is therefore a need to conduct research that can inform interventions to improve treatment success rate particularly in sub Saharan Africa where the burden of tuberculosis and HIV are both very high. The burden of tuberculosis is higher among people living with Human Immunodeficiency Virus (PLHIV) where it is the number one cause of mortality [7]. Accordingly, more data are needed especially from rural or remote areas on treatment outcomes among persons with tuberculosis to inform interventions.

Therefore, the purpose of this study was to measure treatment outcomes, namely treatment success and mortality rates, among persons with BC-PTB in rural eastern Uganda, determine whether HIV infection is associated with these treatment outcomes, and identify other factors amenable to interventions that can contribute to treatment success rate.

## Methods

### Study design

We retrieved and reviewed records for adult persons with BC-PTB, persons with a biological specimen that is positive for *Mycobacterium tuberculosis* (MTB) on smear microscopy, culture, or molecular test like GeneXpert. We considered the 10 largest tuberculosis diagnostic and treatment units in the districts of Soroti, Kumi, Ngora, and Serere, all in eastern Uganda for data review and retrieval. The records are routinely collected by the TB clinics for use in reporting case load, tracking patient outcomes and form part of the National TB and Leprosy program surveillance. The records capture demographics, laboratory and clinical outcomes.

### Variables and measurements

The independent variables included the following: district where the tuberculosis patient received treatment, level and location of health facility, health facility ownership type, year of tuberculosis treatment initiation, sex, age category, type of tuberculosis patient (new or previously treated), transfer-in status, baseline *MTB* load, type of drug regimen, HIV sero-status, type of Directly Observed Therapy Short Course (DOTS), availability of a treatment supporter, and patient residence. The treatment outcome data included: cured, treatment completed, treatment failed, lost to follow-up, died, transferred out, treatment success, and not evaluated.

All participants classified as lost to follow-up were verified by the TB focal persons of respective study sites using either the district TB unit or HIV register, while those reported as dead were confirmed by home visits and presentation of death certificates by either a treatment supporter, Community Health Worker, or family member.

The primary outcome of the analysis was treatment success rate and the secondary was mortality rate, both defined according to the WHO criteria [8]. We computed treatment success rate as the percentage of adult BC-PTB cases registered under DOTS in a given year who completed tuberculosis treatment with bacteriologic evidence of success (cured) or not cured but had completed treatment. Mortality rate was computed as the percentage of adult persons with BC-PTB who died from any cause during tuberculosis treatment.

### Inclusion and exclusion criteria

We included all adult (15 years and older) persons with BC-PTB, diagnosed and treated between January 2015 and June 2018. The participants were either persons with new BC-PTB diagnosis or previously treated persons with BC-PTB, and if they were transferred-in, this should not have happened after 2 months of treatment at the preceding health facilities. Participants in this retrospective cohort were followed from the time of treatment initiation to treatment completion, with an average follow-up of 6 to 8 months. This study excluded participants with no treatment outcome evaluation namely those who were transferred out to other health facilities and those whose treatment outcome was unknown to the reporting TB unit.

### Data analysis

In univariate analysis, we computed frequencies and percentages for categorical variables, and means with standard deviations for numerical data. In the bivariate analysis, we assessed differences in observed and expected frequencies in categorical variables between participants with successful versus non-successful tuberculosis treatment, and between persons with BC-PTB who died versus those who survived using the Chi-squared test for expected cell counts above five, or the Fisher’s exact test for expected cell counts less than five. For numeric data, we used Student’s t-test to assess differences in their means. We performed sensitivity analysis to examine the effect of excluding persons with BC-PTB diagnosed by GeneXpert since they did not have bacilli load data.

We used modified Poisson regression analysis with robust standard errors to perform multivariate analysis for all statistically significant variables identified at the bivariate analysis and reported the results as risk ratios (RR). The RR was a better measure of effect over the odds ratio (OR) for two reasons: 1) Our outcome variables had prevalence greater than 10%, which we considered as a frequent outcome, the OR overestimates the degree of association compared to the RR; 2) When an outcome is rare (less than 10%), both RR and OR estimates are comparable [9]. Since one of the outcome was large and another was less frequent, the RR provides an unbiased measure of effect and ensures harmonized reporting. We reported each RR with the corresponding 95% confidence interval (CI), for both the unadjusted and adjusted results. The data analysis was performed in R programing language and statistical software version 3.5.2 [10] at the 5% level of significance.

### Human subjects’ issues and ethics approval

This study was reviewed and approved by Mbarara University of Science and Technology Research Ethics Committee (Reference number 03/11–18), and the Uganda National Council for Science and Technology (Reference number HS 2531). The need for patient consent was waived by the ethics committee because data collection involved retrieval of records from large numbers of persons with BC-PTB, for whom it would have been logistically impractical to reach and seek individual consent. Data were handled confidentially and anonymized by excluding personal identifiers namely names and locations at the time of data abstraction.

## Results

### Study profile of persons with BC-PTB in eastern Uganda

**Fig. 1:**
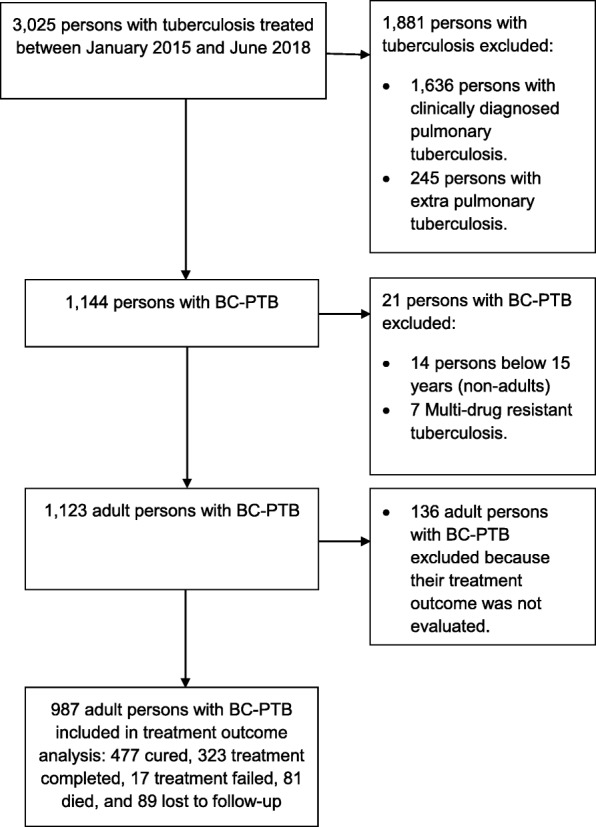
Study profile for treatment outcome analysis for adult persons with bacteriologically confirmed pulmonary tuberculosis in rural eastern Uganda

Of 3025 persons with TB treated between January 2015 and June 2018 shown in Fig. 1, we excluded with reasons: 1) 1881 persons without BC-PTB of whom 1636 were persons with clinically diagnosed TB and 245 were persons with extra-pulmonary TB; 2) 14 persons with BC-PTB because they were below 15 years of age, and; 3) seven persons with BC-PTB because they had multi-drug resistant TB. We therefore retrieved records for 1123 adult persons with BC-PTB and further excluded 136 persons with BC-PTB with no treatment outcome evaluation either because they were transferred to another health facility or their treatment outcome was unknown to the reporting health facility at the time of data abstraction. Therefore, our subsequent analyses included 987 records for adult persons with BC-PTB.

### Characteristics of participants with successful treatment outcome in eastern Uganda

We presented and compared the characteristics of participants with and without treatment success and the results are shown in Table 1. Participants who were successfully treated were on average younger than those who were unsuccessfully treated: 37.89 (SD = 15.15) years versus 41.20 (SD = 15.70), *p* = 0.008. Most of the successfully treated participants were males (510 (63.7%)), aged 15 to 34 years (387 (48.4%)), and persons with new BC-PTB diagnosis (708 (88.5%)). Other participant characteristics namely sex, age, and HIV status differed between those successful treated and those unsuccessfully treated.

**Table 1:** Baseline characteristics of successfully and unsuccessfully treated participants

Characteristics	Level	Tuberculosis treatment outcome		***P***-value
		Unsuccessful (No. (%))	Successful (No. (%))	
All participants		187 (18.9)	800 (81.1)	
District	Soroti	104 (55.6)	359 (44.9)	< 0.001
	Kumi	49 (26.2)	179 (22.4)	
	Ngora	26 (13.9)	115 (14.4)	
	Serere	8 (4.3)	147 (18.4)	
Level of health facility	Health Center IV	67 (35.8)	341 (42.6)	0.221
	General Hospital	40 (21.4)	161 (20.1)	
	Referral Hospital	80 (42.8)	298 (37.2)	
Location of health facility	Rural	45 (24.1)	230 (28.7)	0.232
	Urban	142 (75.9)	570 (71.2)	
Type of health facility ownership	Public	173 (92.5)	707 (88.4)	0.132
	Private not-for-profit	14 (7.5)	93 (11.6)	
Year of tuberculosis treatment	2015	69 (36.9)	298 (37.2)	0.322
	2016	45 (24.1)	172 (21.5)	
	2017	47 (25.1)	176 (22.0)	
	2018	26 (13.9)	154 (19.2)	
Sex	Female	46 (24.6)	290 (36.2)	0.003
	Male	141 (75.4)	510 (63.7)	
Age category	15–34	73 (39.0)	387 (48.4)	0.037
	35–50	66 (35.3)	262 (32.8)	
	More than 50	48 (25.7)	151 (18.9)	
Age (mean (SD))		41.20 (15.70)	37.89 (15.15)	0.008
Type of patient	New	154 (82.4)	708 (88.5)	0.031
	Previously treated	33 (17.6)	92 (11.5)	
Pre-therapy *MTB* load	1+	25 (13.4)	117 (14.6)	0.634
	2+	43 (23.0)	215 (26.9)	
	3+	64 (34.2)	251 (31.4)	
	Diagnosis by GeneXpert	55 (29.4)	217 (27.1)	
Drug regimen	2RHZE/4RH	105 (56.1)	496 (62.0)	0.172
	2RHZE/6HE	63 (33.7)	250 (31.2)	
	2RHZES/1RHZE/5RHE	19 (10.2)	54 (6.8)	
HIV status	Negative	113 (60.4)	591 (73.9)	< 0.001
	Positive	74 (39.6)	209 (26.1)	
Type of Directly Observed Therapy Short Course	Health facility	12 (6.4)	33 (4.1)	0.247
	Community	175 (93.6)	767 (95.9)	
Treatment supporter availability	No	24 (12.8)	98 (12.2)	0.924
	Yes	163 (87.2)	702 (87.8)	
Living in the same sub-county as that of the health facility	Yes	60 (39.5)	312 (45.4)	0.214
	No	92 (60.5)	375 (54.6)	

Note: 1) 2RHZE/4RH: 2 months of Rifampicin (R), Isoniazid (H), Pyrazinamide (Z),Ethambutol / 4 months of RH; 2) 2RHZE/6EH: 2 months of RHZE)/ 6 months of EH; 3) 2RHZES/IRHZE/5RHE: 2 months of RHZE, Streptomycin/One month of RHZE/ 5 months of RHE; 4) MTB: *Mycobacterium tuberculosis*

There was no difference in treatment success rate with respect to health facility attributes (level, location, and ownership type), year of treatment, drug regimen and baseline bacilli. Sensitivity analysis results showed that the exclusion of persons with BC-PTB diagnosed by GeneXpert resulted in no change in proportions of treatment success (Chi-square test, *p* value changed from 0.634 to 0.511).

### Distribution of treatment outcomes among persons with BC-PTB in eastern Uganda

In Table 2, the treatment outcomes for the 1123 records were as follows: 477 (42.5%) cured, 323 (28.8%) treatment completed, 17 (1.5%) treatment failed, 81 (7.2%) died, 89 (7.9%) lost to follow-up, and 136 (12.1%) not evaluated. Table 2 also shows the treatment outcomes stratified by district of assessment. After excluding not evaluated participants, revised analysis showed that 800 (81.1%) persons with BC-PTB were successfully treated.

**Table 2:** Study profile and tuberculosis treatment outcomes by district in eastern Uganda

**Characteristics**	**Soroti** ***n*** **= 541**	**Kumi** ***n*** **= 279**	**Ngora** ***n*** **= 148**	**Serere** ***n*** **= 155**	**Total** ***n*** **= 1123**
**Treatment outcomes (*n*** **= 1123)**	**n (%)**	**n (%)**	**n (%)**	**n (%)**	**n (%)**
Cure	247 (45.7)	122 (43.7)	31 (20.9)	77 (49.7)	477 (42.5)
Treatment completed	112 (20.7)	57 (20.4)	84 (56.8)	70 (45.2)	323 (28.8)
Treatment failed	10 (1.8)	6 (2.2)	0 (0.0)	1 (0.6)	17 (1.5)
Died	45 (8.3)	15 (5.4)	16 (10.8)	5 (3.2)	81 (7.2)
Lost to follow-up	49 (9.1)	28 (10.0)	10 (6.8)	2 (1.3)	89 (7.9)
Not evaluated	78 (14.4)	51 (18.3)	7 (4.7)	0 (0.0)	136 (12.1)
**Tuberculosis treatment outcome available (*n*** **= 987)**	**Soroti** ***n*** **= 463**	**Kumi** ***n*** **= 228**	**Ngora** ***n*** **= 141**	**Serere** **n = 155**	**Total** **n = 987**
Unsuccessful	104 (22.5)	49 (21.5)	26 (18.4)	8 (5.2)	187 (18.9)
Successful	359 (77.5)	179 (78.5)	115 (81.6)	147 (94.8)	800 (81.1)

Note: In computation of mortality and treatment success, participants with missing treatment outcome (*n* = 136) were excluded

### Factors associated with treatment success among persons with BC-PTB in eastern Uganda

Table 3 is a summary of results at unadjusted and adjusted analyses. In unadjusted analysis, participants from Serere district had increased treatment success rate compared to those from Soroti district (RR, 1.22; 95% CI, 1.15–1.30). However, treatment success rate was lower among males compared to females (RR, 0.91; 95% CI, 0.86–0.96), those more than 50 years of age than 15 to 34 years of age (RR, 0.88; 95% CI, 0.81–0.97), previously treated persons with BC-PTB compared to persons with new BC-PTB diagnosis (RR, 0.90; 95% CI, 0.80–1.00), and HIV infected than HIV non-infected (RR, 0.88; 95% CI, 0.81–0.95). When we adjusted for all statistically significant factors, treatment success was less likely to occur among males (aRR, 0.92; 95% CI, 0.87–0.98), patients older than 50 years of age (aRR, 0.89; 95% CI, 0.81–0.97), those HIV infected (aRR, 0.88; 95% CI, 0.82–0.95) but more among residents from Serere compared to Soroti district (aRR, 1.22; 1.14–1.30).

**Table 3:** Factors associated with treatment success among persons with BC-PTB in eastern Uganda

Characteristics	Level	Tuberculosis treatment outcome		Modified Poisson regression analysis			
		Unsuccessful (No. (%))	Successful (No. (%))	Unadjusted analysis		Adjusted analysis	
Participants		187 (18.9)	800 (81.1)	RR	95% CI	aRR	95% CI
District	Soroti	104 (55.6)	359 (44.9)	Ref		Ref	
	Kumi	49 (26.2)	179 (22.4)	1.01	(0.93,1.10)	1.02	(0.94,1.11)
	Ngora	26 (13.9)	115 (14.4)	1.05	(0.96,1.15)	1.04	(0.95,1.14)
	Serere	8 (4.3)	147 (18.4)	1.22^***^	(1.15,1.30)	1.22^***^	(1.14,1.30)
Sex	Female	46 (24.6)	290 (36.2)	1		1	
	Male	141 (75.4)	510 (63.7)	0.91^**^	(0.86,0.96)	0.92^**^	(0.87,0.98)
Age group	15–34	73 (39.0)	387 (48.4)	1		1	
	35–50	66 (35.3)	262 (32.8)	0.98	(0.92,1.05)	0.97	(0.91,1.04)
	More than 50	48 (25.7)	151 (18.9)	0.88^**^	(0.81,0.97)	0.89^**^	(0.81,0.97)
Persons with BC-PTB	New	154 (82.4)	708 (88.5)	1		1	
	Previously treated	33 (17.6)	92 (11.5)	0.90^*^	(0.80,1.00)	0.94	(0.84,1.05)
HIV status	Negative	111 (60.0)	583 (73.6)	1		1	
	Positive	74 (40.0)	209 (26.4)	0.88^**^	(0.81,0.95)	0.88^**^	(0.82,0.95)

Note: 95% confidence intervals for risk ratio (RR) in brackets; ^*^p < 0.05, ^**^p < 0.01, ^***^p < 0.001; RR: Unadjusted risk ratio; aRR: Adjusted risk ratio

### Baseline characteristics of persons with BC-PTB by survival status in eastern Uganda

Table 4 shows the distribution of participant characteristics stratified by survival, namely alive or died. Of 987 participants whose treatment outcomes were evaluated, 81 (8.2%) died. Most of the deaths were at referral hospital level (40/81 or 49.4%) and in 2015 (35/81 or 43.2%). Participants who died were on average older than those who were alive: 44.80 ± 16.82 versus 37.95 ± 15.05 years, *p* < 0.001. There was a statistically significant difference in mortality rate based on the district where treatment was received, level of health facility, year of tuberculosis treatment, participants’ age, participant HIV sero-status, baseline bacilli, and form of DOTS. In sensitivity analysis, we found the exclusion of persons with BC-PTB diagnosed by GeneXpert resulted in no change in proportions of mortality (Chi-square test, *p* value changed from 0.948 to 0.887).

**Table 4:** Baseline characteristics of participants by survival status in eastern Uganda

Characteristics	Level	Alive *n* = 906 (91.8%)	Died *n* = 81 (8.2%)	*p*-value
District	Soroti	418 (46.1)	45 (55.6)	0.027
	Kumi	213 (23.5)	15 (18.5)	
	Ngora	125 (13.8)	16 (19.8)	
	Serere	150 (16.6)	5 (6.2)	
Level of health facility	Health Center IV	386 (42.6)	22 (27.2)	0.023
	District Hospital	182 (20.1)	19 (23.5)	
	Referral Hospital	338 (37.3)	40 (49.4)	
Location of health facility	Rural	252 (27.8)	23 (28.4)	1.000
	Peri-urban	654 (72.2)	58 (71.6)	
Health facility ownership	Public	808 (89.2)	72 (88.9)	1.000
	Private not for profit	98 (10.8)	9 (11.1)	
Year of TB treatment	2015	332 (36.6)	35 (43.2)	0.022
	2016	192 (21.2)	25 (30.9)	
	2017	209 (23.1)	14 (17.3)	
	2018	173 (19.1)	7 (8.6)	
Age category (years)	15–34	436 (48.1)	24 (29.6)	0.002
	35–50	297 (32.8)	31 (38.3)	
	> 50	173 (19.1)	26 (32.1)	
	Mean (SD)	37.95 (15.05)	44.80 (16.82)	< 0.001
Sex	Male	593 (65.5)	58 (71.6)	0.319
	Female	313 (34.5)	23 (28.4)	
Persons with BC-PTB	New	792 (87.4)	70 (86.4)	0.933
	Previously treated	114 (12.6)	11 (13.6)	
Transfer in	No	830 (91.6)	73 (90.1)	0.801
	Yes	76 (8.4)	8 (9.9)	
Pre-therapy *MTB* load	1+	130 (14.3)	12 (14.8)	0.948
	2+	238 (26.3)	20 (24.7)	
	3+	287 (31.7)	28 (34.6)	
	GeneXpert	251 (27.7)	21 (25.9)	
Drug regimen	2RHZE/4RH	560 (61.8)	41 (50.6)	0.136
	2RHZE/6HE	280 (30.9)	33 (40.7)	
	2RHZES/1RHZE/5RHE	66 (7.3)	7 (8.6)	
HIV status	Negative	673 (74.3)	31 (38.3)	< 0.001
	Positive	233 (27.7)	50 (61.7)	
Type of Directly Observed Therapy Short Course	Health facility	36 (4.0)	9 (11.1)	0.008
	Community	870 (96.0)	72 (88.9)	
Treatment support availability	No	111 (12.3)	11 (13.6)	0.864
	Yes	795 (87.7)	70 (86.4)	

Note: 1) 2RHZE/4RH: 2 months of Rifampicin (R), Isoniazid (H), Pyrazinamide (Z),Ethambutol / 4 months of RH; 2) 2RHZE/6EH: 2 months of RHZE)/ 6 months of EH; 3) 2RHZES/IRHZE/5RHE: 2 months of RHZE, Streptomycin/One month of RHZE/ 5 months of RHE; 4) *MTB: Mycobacterium tuberculosis*

### Factors associated with mortality among persons with BC-PTB in eastern Uganda

Table 5 presents results for factors associated with mortality rate. In the unadjusted analysis, mortality rate was lower among participants who received treatment under community-based DOTS than facility-based DOTS (RR, 0.38; 95% CI, 0.20–0.71). However, mortality was more likely to occur when treatment of tuberculosis was given at a Referral Hospital compared to a Health Center IV level (RR, 1.96; 95% CI, 1.19–3.24), when persons with BC-PTB were older than 50 years of age compared to 15 to 34 years (RR, 2.50; 95% CI, 1.47–4.25), and when persons with BC-PTB were HIV infected compared to HIV non-infected (RR, 4.01; 95% CI, 2.62–6.15). In adjusted analysis, mortality was lower when tuberculosis treatment was initiated in the years after 2015, with a 20% reduction for every 1 year lapse from 2015 up to 2018 when the last data were retrieved (aRR, 0.80; 95% CI, 0.66–0.97). Delivery of tuberculosis treatment under community-based DOTS was protective of mortality (aRR, 0.26; 95% CI, 0.13–0.50). But mortality rate was higher among persons with BC-PTB aged 50 years and older (aRR, 2.93; 95% CI, 1.74–4.92) and those who were HIV infected (aRR, 4.48; 95% CI, 2.95–6.79).

**Table 5:** Factors associated with mortality among persons with BC-PTB in rural eastern Uganda

Characteristics	Level	Alive ***n*** = 906 (91.8%)	Died ***n*** = 81 (8.2%)	Modified Poisson regression analysis			
				Unadjusted analysis (RR, 95% CI)		Adjusted analysis (RR, 95% CI)	
District	Soroti	418 (46.1)	45 (55.6)	Ref		Ref	
	Kumi	213 (23.5)	15 (18.5)	0.68	(0.39,1.19)	0.74	(0.25,2.18)
	Ngora	125 (13.8)	16 (19.8)	1.17	(0.68,2.00)	1.74	(0.63,4.82)
	Serere	150 (16.6)	5 (6.2)	0.33^*^	(0.13,0.82)	0.57	(0.18,1.88)
Level of health facility	Health Center IV	386 (42.6)	22 (27.2)	Ref		Ref	
	District Hospital	182 (20.1)	19 (23.5)	1.75	(0.97,3.16)	1.38	(0.69,2.75)
	Referral Hospital	338 (37.3)	40 (49.4)	1.96^**^	(1.19,3.24)	1.99	(0.81,4.89)
Year of TB treatment	2015			Ref		Ref	
	One year increase			0.78^**^	(0.64,0.93)	0.80^*^	(0.66,0.97)
Age group	15–34	436 (48.1)	24 (29.6)	Ref		Ref	
	35–50	297 (32.8)	31 (38.3)	1.81^*^	(1.08,3.03)	1.56	(0.96,2.55)
	> 50	173 (19.1)	26 (32.1)	2.50^***^	(1.47,4.25)	2.93^***^	(1.74,4.92)
HIV status	Negative	673 (74.3)	31 (38.3)	Ref		Ref	
	Positive	233 (27.7)	50 (61.7)	4.01***	(2.62,6.15)	4.48^***^	(2.95,6.79)
Type of DOTS	Facility	36 (4.0)	9 (11.1)	Ref		Ref	
	Community	870 (96.0)	72 (88.9)	0.38^**^	(0.20,0.71)	0.26^***^	(0.13,0.50)

Note: 95% confidence intervals for risk ratio (RR) in brackets; ^*^p < 0.05, ^**^p < 0.01, ^***^p < 0.001; RR: Unadjusted risk ratio; aRR: Adjusted risk ratio

## Discussion

Our data from rural eastern Uganda shows that treatment success rate for persons with BC-PTB is suboptimal and mortality rate is high. Our data also shows that HIV infection and older age are associated with reduced treatment success rate and increased mortality rate while CB-DOTS is associated with reduced mortality compared to facility-based DOTS. The treatment success rate reported here is no different from that reported in a recent meta-analysis of tuberculosis treatment success rate for sub Saharan Africa, which was 76.2% [6]. Since the treatment success rate in eastern Uganda falls below the desired target of at least 90%, site-specific measures are needed to address barriers to achieving high treatment success rate.

Mortality rate was high in this cohort of adult persons with BC-PTB. We were not able to ascertain the exact date of death from the records, therefore it is not clear whether mortality occurred early or later during treatment. The mortality rate in this study is comparable to that observed in other cohorts of persons with tuberculosis in sub Saharan Africa such as Ethiopia [11]. However, some cohorts from sub Saharan Africa have reported significantly lower mortality rates of less than 5% [12, 13]. It is clear that a wide variation in mortality rates in the treatment cohorts in sub Saharan Africa exists. These differences may be explained by the prevalence of HIV in the different countries across the continent, especially given the relationship between HIV and mortality [14] and that we found in our cohort.

Our study shows HIV infected persons with BC-PTB have reduced treatment success rate and increased mortality rate. These findings are not unique because the relationship between HIV and tuberculosis is well-established [15]. HIV is a known strong risk factor for tuberculosis disease, alters the clinical presentation, progression, and prognosis of tuberculosis as well as response to treatment [14]. In addition, tuberculosis disease is the commonest opportunistic infection among PLHIV, and the leading cause of mortality [7]. Dual treatment of tuberculosis and HIV is associated with increased pill burden and this may lead to medication fatigue and potentially non-adherence [16, 17]. Non-adherence to anti-tuberculosis medications in turn reduce treatment success rate [18] and increase mortality rate [19]. In general, several pathways could explain the observed relationship. Our finding is consistent with several studies in sub Saharan Africa [20–28] where HIV infected persons have been documented to have reduced treatment success rate compared to HIV uninfected persons. HIV infected persons with BC-PTB might hence benefit from closer clinical and laboratory monitoring and treatment adherence support so as to improve treatment success rate and reduce mortality rate.

In practice, strengthening the collaboration between tuberculosis and HIV control programs would improve the management of HIV infected persons with tuberculosis. This might enhance treatment success rate and reduced mortality rate.

We found that persons with BC-PTB who were more than 50 years of age had reduced treatment success rate and increased mortality rate. Studies in several parts of sub Saharan [20, 22, 23, 25, 27, 29–33], indicate that older or increasing age is associated with reduced treatment success rate among persons with tuberculosis. This could be because older persons interrupt treatment adherence more often than younger persons, are challenged by several health determinants such as low socio-economic status as well as low immunity that cannot effectively fight infections [25], and medical complications that comes along with ageing. Comparable to our findings, a systematic review [34] found that persons who are 30 years of age and beyond tend to be non-adherent to anti-tuberculosis medications. Older persons with tuberculosis might therefore benefit from close monitoring for response to tuberculosis treatment [35].

Our study indicates that male sex is associated with lower treatment success rate compared to female sex. This finding is consistent with several studies in Africa [22, 25, 27]. In particular, the study in Nigeria [27], reports gender disparities in treatment outcomes where male sex was associated with reduced sputum smear conversion at 2 and 5 months of treatment, increased treatment failure and reduced treatment success rate. Another study in Benin reports male sex is associated with increased treatment failure [36], and treatment failure translates into unsuccessful treatment of tuberculosis.

The observed difference could be attributed to men’s poor healthcare seeking behavior compared to women [37], which translates to delayed diagnosis of tuberculosis and missing of clinic visits for drug refills hence treatment non-adherence and ultimately unsuccessful treatment of tuberculosis. Our finding implies that the need to design gender-specific interventions across tuberculosis programs might be beneficial.

Our data shows lower mortality rate in recent years, between 2016 and 2018, compared to later year. This improvement may be attributable to improvements in healthcare system over time: better staffing and improved healthcare provider competence and confidence to manage persons with BC-PTB through mentorships and coaching, and better counseling among others. Elsewhere [38], healthcare providers indicated that tuberculosis clinics have been established across most health facilities to provide more time to understand and solve the challenges of persons with tuberculosis. This approach has led to better management of persons with tuberculosis. In addition, continuous quality improvement interventions have been introduced to tackle operational challenges in tuberculosis programing. It was stated that these initiatives have strengthened the healthcare system.

Our study indicates that community-based DOTS is associated with reduced mortality rate compared to facility-based DOTS. This finding is consistent with the results of previous systematic reviews and meta-analyses where community-based DOTS is reported to improve treatment completion and cure, and to reduce mortality rate [39–42]. One of the plausible reasons is that in community-based DOTS, persons with tuberculosis take medications under the direct supervision of a treatment supporter at home or in their community [43], and this is likely to result into better treatment adherence [44] and completion hence better cure rate and reduced mortality rate.

Our result is also consistent with findings of another meta-analysis which reports community-based DOTS is associated with lower mortality rate compared to facility-based DOTS [45]. Although there is sufficient evidence that community-based DOTS has better treatment outcomes compared to facility-based DOTS [39, 40], our result may suffer confounding due to indication, especially since we did not have data on disease severity. We recommend that in future, studies should measure disease severity when comparing mortality between facility and community-based DOTS.

### Study strengths and limitations

Our study has several strengths. First, our sample size was large. Second, our study is the first in eastern Uganda. It has therefore set the benchmark for further studies in the same locality. Third, all the treatment outcomes were verified and this ensured data analyzed were accurate. However, there are some limitations that should be considered. This study was conducted in a rural setting. The findings may not apply to an urban setting due to variations in socio-economic status and structural challenges associated with rural dwelling such as longer distances to health facilities. Also, our results do not apply to persons with tuberculosis below 15 years of age and those with other forms of tuberculosis. Lastly, our data spanned a period of 3 years, which might not be sufficient to demonstrate long term trends in treatment success and mortality rates.

## Conclusions

In conclusion, we have shown that adult persons with BC-PTB in rural eastern Uganda have suboptimal treatment success rate and high mortality rate. Tuberculosis and HIV co-infection and older age were associated with reduced treatment success and increased mortality rates.

Integration of tuberculosis and HIV prevention and management should be enhanced; particularly early screening and prophylaxis for tuberculosis among HIV infected persons, especially those who are older as they have poorer prognosis with TB disease. Community-based DOTS should be implemented widely and more research should be done to examine successful ways for implementation of Community-based DOTS especially in rural areas such as those where our study was conducted.

## Data Availability

The dataset used/or analyzed during the current study are available from the corresponding author on reasonable request.

## Abbreviations

aRR: Adjusted risk ratio
BC-PTB: Bacteriologically confirmed pulmonary tuberculosis
DOTS: Directly observed therapy short course
HIV: Human immunodeficiency virus
uRR: Unadjusted risk ratio
WHO: World Health Organization
